# Enhanced Crystallization Behaviors of Silicon-Doped Sb_2_Te Films: Optical Evidences

**DOI:** 10.1038/srep33639

**Published:** 2016-09-19

**Authors:** Shuang Guo, Liping Xu, Jinzhong Zhang, Zhigao Hu, Tao Li, Liangcai Wu, Zhitang Song, Junhao Chu

**Affiliations:** 1 Department of Electronic Engineering, East China Normal University, Shanghai 200241, China; 2 State Key Laboratory of Functional Materials for Informatics, Shanghai Institute of Microsystem and Information Technology, Chinese Academy of Sciences, Shanghai 200050, China

## Abstract

The optical properties and structural variations of silicon (Si) doped Sb_2_Te (SST) films as functions of temperature (210–620 K) and Si concentration (0–33%) have been investigated by the means of temperature dependent Raman scattering and spectroscopic ellipsometry experiments. Based upon the changes in Raman phonon modes and dielectric functions, it can be concluded that the temperature ranges for intermediates and transition states are estimated to 150, 120, 90, and 0 K, corresponding to ST, SST25%, SST28%, and SST33% films, respectively. The phenomenon also can be summarized by the thermal evolutions of interband electronic transition energies (*E*_*n*_) and partial spectral weight integral (*I*). The disappearance of intermediate (INT) state for SST33% film between amorphous (AM) and hexagonal (HEX) phases can be attributed to the acceleratory crystallization of HEX phase by Si introduction. It illustrates that the risk of phase separation (Sb and Te) during the cyclic phase-change processes decreases with the increasing Si concentration. The enhanced crystallization behaviors can optimize the data retention ability and the long term stability of ST by Si doping, which are important indicators for phase change materials. The performance improvement has been analyzed qualitatively from the optical perspective.

Phase change materials have been applied into electrical and optical data storage commercially^1^,^2^. The applications, derived from the fast switching properties and tremendous optical contrast between amorphous (*a*-) and crystalline (*c*-) states, include all-photonic memories, optical modulators, solid-state displays, and plasmonic-based circuits, etc. refs 3, 4, 5, 6. The most representative phase change materials are the nucleation-dominant GeTe-Sb_2_Te_3_ compositions and the growth-dominant Sb-based compounds^7^,^8^,^9^,^10^,^11^,^12^,^13^. The composition close to the “eutectic” Sb_2_Te (ST) is considered as a potential candidate in optical data storage because of its fast crystallization speed^14^,^15^. However, the amorphous thermal stability of Sb-Te has been worrying, which has a strong impact on the data retention ability of the material. In the usual way, doping other elements, such as Ge, Al, Cu, Ag, and In, etc. refs 16, 17, 18, 19, 20, into Sb-Te alloys can improve the thermal stability effectively. In recent years, Si has been chosen as a dopant due to its low cost and high efficiency. Si-doped Sb-Te phase change materials have been explored due to its better thermal stability and lower threshold^21^,^22^,^23^,^24^,^25^,^26^.

Among the Si-doped Sb-Te material systems, Si doped Sb_2_Te (SST) has been confirmed with a higher crystallization temperature and a superior thermal stability of the amorphous phase, as well as a better data retention ability by the means of electrical and thermal characterization methods^23^,^24^. The crystal structure of SST films has been examined by transmission electron microscopy (TEM) and X-ray diffraction (XRD), which is consisted of amorphous (AM) Si and crystalline Sb_2_Te with hexagonal (HEX) phase. The grain size of SST decreases with the increasing Si concentration, forming a more uniform grain distribution^24^. Note that the Sb_2_Te with hexagonal unit cell contains five-layer atom stacks of Sb_2_Te_3_ and two-layer atom stacks of Sb_2_^27^. However, for previous studies, the investigation of crystal structure for SST were basically by testing the samples after rapid thermal annealing, which only can confirm the final crystalline phase of SST. The accurate structural transformation during the crystallization process is ambiguous. Moreover, up to now, the studies for SST mostly focused on electrical and thermal properties. While the optical properties for SST were ignored and scarcely investigated, which is especially significant to comprehend the information on the structure and dynamics of the phase change process. In addition, although the optimized data retention ability and the long term stability of ST by Si doping have been determined by electrical methods, the essence of performance improvement needs further study. Therefore, it is necessary to provide additional insight into the underlying mechanism of the crystallization behaviors for SST.

To fill the gaps of current studies, in this work, the optical properties and structural variations of Si-doped ST have been investigated by the means of temperature dependent Raman scattering and spectroscopic ellipsometry (SE) experiments. The structural transformation during the thermally-induced crystallization process has been systematic studied by analyzing the evolution of Raman spectra with the temperature. Besides, we elucidate the enhanced crystallization behaviors of SST films, namely, the disappearance of intermediate (INT) state of SST 33%, which can be concluded by analyzing the crystal and electronic structures. Furthermore, the lattice dynamics, optical constants, and electronic transitions of SST films as functions of temperature and Si concentration have been discussed in detail.

## Results and Discussion

### Raman scattering

**Figure 1:**
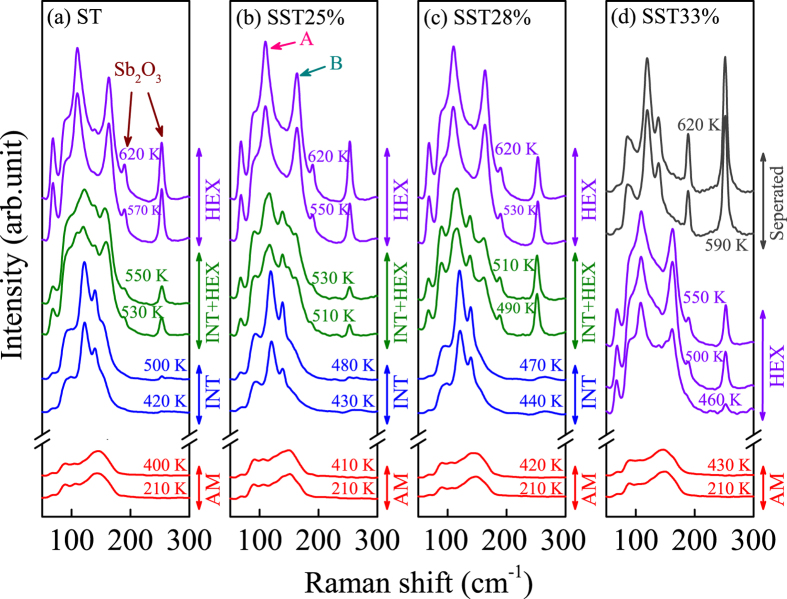
Temperature dependent Raman spectra of (**a**) ST, (**b**) SST25%, (**c**) SST28%, and (**d**) SST33% films from 210 to 620 K, respectively. Note that the two Raman peaks located at around 189 cm^−1^ and 253 cm^−1^ correspond to the oxide phase of Sb_2_O_3_. Label A and B represent the two main Raman peaks of ST and SST films in HEX phase.

Raman spectroscopy can provide significant information on structural variation and lattice vibrations. Figure 1 displays the Raman spectra of ST and SST films recorded in the temperature range from 210 to 620 K. For AM films, there is a wide broadening band ranging from 60 to 180 cm^−1^. The Raman spectra of ST exhibit three obvious variations with increasing temperature Fig. 1(a). Similar crystallization behaviors can be concluded for SST25% and SST28%, as depicted in Fig. 1(b,c), respectively. The crystallization temperature (AM-INT/HEX) is elevated from 410 to 440 K with the Si concentration increasing to 33%. It indicates that Si doping inhibits the crystallization, which is in accordance with previous studies^21^,^24^. The phase change temperature for the basic formation of the HEX phase is estimated at 560, 540, and 520 K, corresponding to ST, SST25%, and SST28% films, respectively. It can be found that the intermediates and transition states disappear in SST33% film (Fig. 1(d)). The phenomenon indicates that Si dopants can promote the crystallization of HEX phase for Sb_2_Te film during the process of heating up. Moreover, it illustrates that the possibility of phase separation (Sb and Te) during the cyclic phase-change processes decreases with the increasing Si concentration. The enhanced crystallization behavior is helpful to improve the data retention ability and long term stability of ST by Si doping, which are very crucial factors for phase change materials. In addition, serious phase separation can be discovered with the temperature rising to 590 K, forming the complicated *c*-(Sb + Te) + Sb_2_O_3_. The two peaks at about 189 and 253 cm^−1^ are the vibrational modes of Sb_2_O_3_^28^. The oxidization of Sb at higher temperatures can be attributed to the existence of a small amount of oxygen in the protective gas, namely, the nitrogen. Note that the two Sb_2_O_3_ peaks cannot be observed in INT state, and the oxidation degree improves with the increasing temperature.

**Figure 2:**
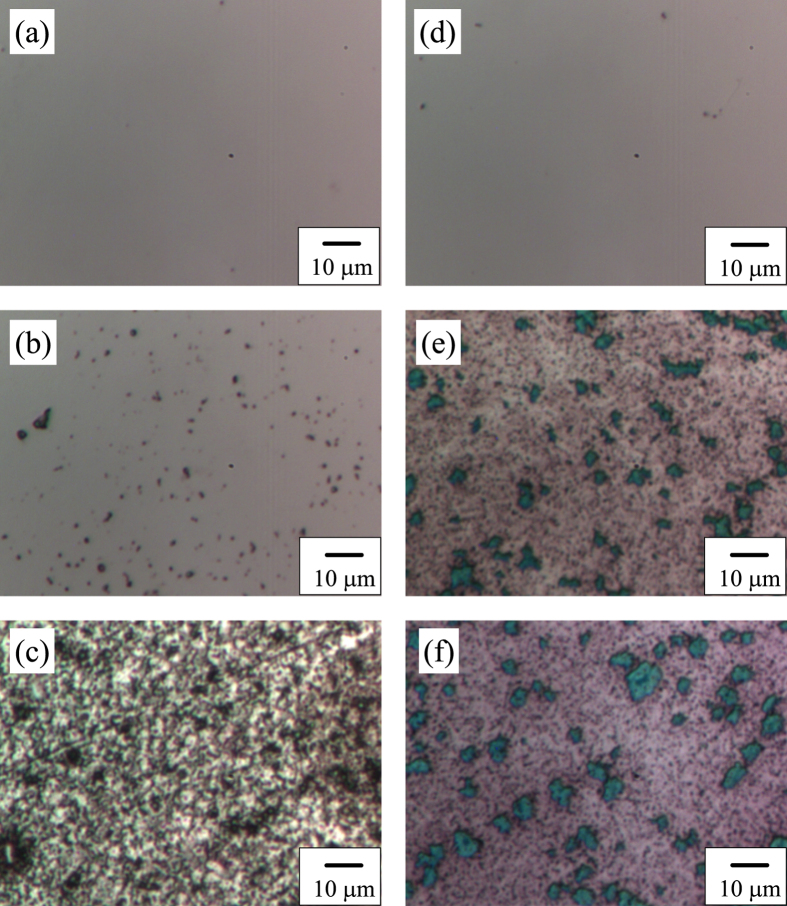
Surface morphologies of ST and SST33% films. (**a–c**) are images of ST film at 300 (AM), 450 (INT), and 560 K (HEX), respectively. (**d–f**) are images of SST33% film at 300 (AM), 450 (HEX), and 560 K (HEX), respectively.

The amorphous structure, intermediate state, and HEX phases for ST and SST films also can be distinguished visually by the change in the surface morphology during Raman scattering experiments. The surface morphologies of ST and SST33% films in different crystal structures were recorded through the 50× microscope. Figure 2 shows the surface morphologies of the ST and SST33% films at 300, 450, and 560 K during the heating process. Figure 2(a,d) are smooth surfaces of amorphous ST and SST33% films, respectively. As the temperature rising to about 410 K, the ST film starts to crystallization gradually. The surface morphology of ST film at 450 K is shown in Fig. 2(b), which corresponds to the INT state. With further heating up, it is noteworthy that the surface morphology of ST film changed significantly near 560 K. The phenomenon can be associated with the basic formation of the HEX phase for ST. Figure 2(c) displays the surface morphology of HEX ST film at 560 K. However, only one apparent change of the surface morphology for SST33% film can be observed at about 440 K with the temperature rising to 620 K, which corresponds to the crystallization from AM to HEX phase. The similar surface morphologies of SST33% film in HEX phase at 450 and 560 K are depicted in Fig. 2(e,f), respectively. The phase change process can be identified directly and real-timely by the evolution of surface morphologies with the increasing temperature.

In order to identify the complex components for INT and HEX states and the Si doping effects on ST film, the Raman spectra were decomposed into a series of bands with the assumption of Lorentz-Gauss line shape approximation. Raman spectra of ST and SST films at 450, 520, and 560 K, as well as the well-fitted deconvolution peaks for ST film are given (see Supplementary Fig. S1). It can be summarized that the ST in INT state is composed of crystalline Sb and Te. Besides, in the final HEX geometry, a thimbleful of crystalline Sb and Te still exist due to the slight incomplete recrystallization caused by the slow heating rate^29^,^30^. Note that no additional peaks can be found in Raman spectra after Si doping. Therefore, no crystalline silicon produced during the crystallization process. From previous studies, it is highly possible that a very few Si atoms bond with Sb or Te after the crystallization. Vast majority Si atoms in SST films are not involved in the phase change process, which is still holding AM phase in the crystalline SST and destroying the long range order lattice of crystal grains^21^,^24^.

**Figure 3:**
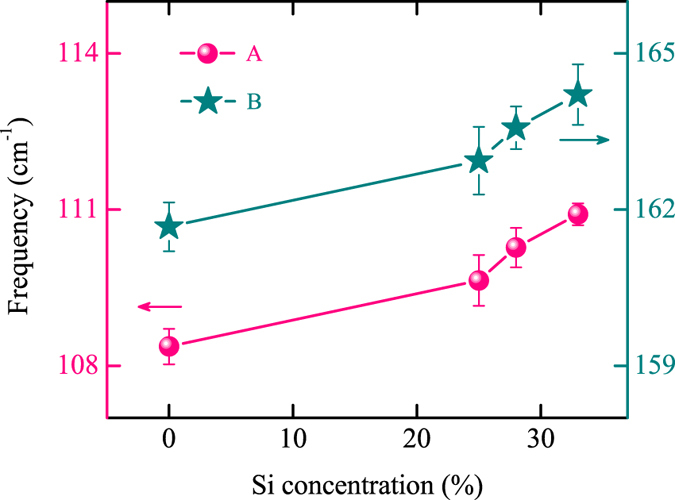
Frequency variations of the two main Raman peaks (A,B) in HEX geometry as a function of Si concentration.

The frequencies of phonon modes for ST and SST films at 450 (INT) and 560 K (HEX) are given (see Supplementary Table S1). The assignments and corresponding frequencies of the Raman phonon modes for ST are consistent with previous study^29^. Although the number and approximate positions of these peaks are the same in the spectra of the corresponding geometries for all four samples, the shift towards lower wave numbers of all the peaks can be extracted with increasing Si concentration. The phenomenon also can be concluded from the original Raman spectra, as shown in Fig. 3. It suggests that the two main Raman peaks marked with A and B (see Fig. 1(b)) in HEX geometry shift from approximately 108 to 111 cm^−1^ and 161 to 164 cm^−1^ with the Si concentration increasing to 33%, respectively. The shift towards lower wave numbers of Raman peaks for SST films with increasing Si concentration can be explained by the compression stress induced by Si doping. The compression is originated from the interface between ST domain boundaries and the separated Si^31^.

### Spectroscopic ellipsometry

The complex optical functions of ST and SST were investigated by temperature dependent SE, which are intrinsically associated with the energy band structures and electronic transitions. The experimental data measured by ellipsometry are the ratio 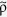 as functions of Ψ and Δ, which can be defined as 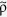 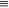 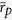/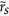 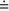 tanΨ*e*^*i*Δ^. Here, 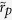 and 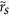 are the reflection coefficient of the polarized lights, which are parallel and perpendicular to the incidence plane angle, respectively. The parameter 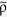 is the functions of incident angle, photon energy, and dielectric functions (ε) of the materials. The dielectric functions can be written as: 〈
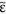
〉 = 〈
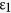
〉+i〈
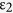
〉 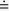 sin^2^ θ [1 + tan^2^ θ (1 − 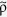)^2^/(1 + 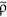)^2^. Here, θ is the incident angle. Note that the dielectric functions are extracted for the material with idealized surface. The imaginary part of the complex optical functions can reflect the electronic transition information of the materials. In the present work, a five-layered model (air/surface rough layer/SST/SiO_2_/Si) was used to extract the dielectric functions of ST and SST films with the WVASE32 software package (J. A. Woollam Co., Inc.). The surface rough layer was defined by effective medium approximation (50% void and 50% film). The dielectric functions were obtained by single Tauc-Lorentz (TL) and Lorentz (L) dispersion models. The imaginary part of TL model can be written as^32^,^33^:


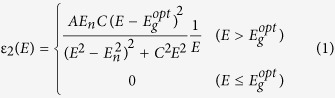


and the real part is given by the Kramers-Krönig transformation (KKT)


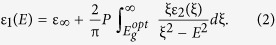


Here, 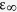 is high frequency dielectric constant, *P* is the Cauchy principal part of the integral, and *E* is incident photon energy. Also, *A*, *E*_*n*_, *C*, and 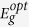 are the amplitude, peak transition energy, broadening term, and Tauc gap energy of the oscillator, respectively. The TL model follows the Kramers-Krönig transformation (KKT), which has been used in semiconductors and dielectric materials successfully^34^. Note that TL model ignores the weak Urbach absorption and localized state. The Lorentz model can be written as:


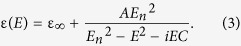


Here, *A*, *E*_*n*_, and *C* are the strength, peak transition energy (center energy), and broadening term of the oscillator, respectively. The Drude-like term for free carriers was added in infrared (IR) region of 0.1–0.6 eV (amorphous and crystalline) and UV-vis region of 0.6–4.13 eV (crystalline). The Drude model can be written as:


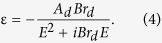


Here, *A*_*d*_ and *Br*_*d*_ are the amplitude and broadening term of the oscillator, respectively^34^,^35^.

**Figure 4:**
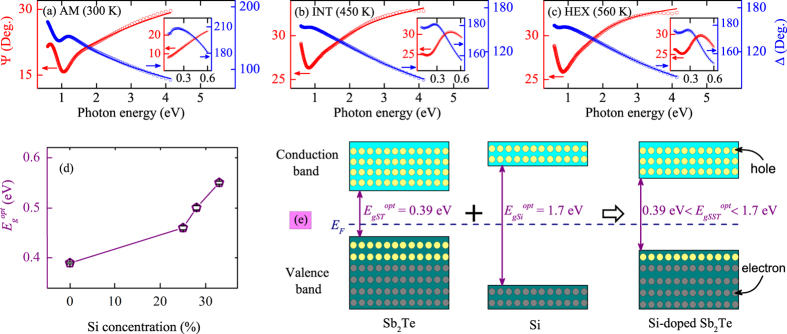
Experimental (dots) and best fitting (solid lines) ellipsometric spectra of Ψ and Δ of ST film for (**a**) AM (300 K), (**b**) INT (450 K), and (**c**) HEX (560 K) structures in UV-vis region of 0.6–4.13 eV, respectively. Note that the insets show the corresponding Ψ and Δ in the IR region of 0.1–0.6 eV. (**d**) The Tauc gap energy (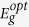) for amorphous films as a function of Si concentration extracted from the Tauc-Lorentz model. (**e**) Schematic representation of the electronic band structure for the amorphous ST, Si, and SST films, where *E*_*F*_ denotes the Fermi energy level.

The dielectric functions of ST and SST films were calculated by fitting the model functions to the measured data. The best fitting parameters for ST and SST films at 300 (AM), 450 (INT), and 560 K (HEX) are given (see Supplementary Table S2). The experimental and best fitting ellipsometric spectra Ψ and Δ of ST film for AM (300 K), INT (450 K), and HEX (560 K) structures are presented in Fig. 4(a–c), respectively. Note that the absorption edge shows an obvious redshift during the change from amorphous to crystalline phase. Figure 4(d) shows the Tauc gap energy (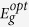) for amorphous films as a function of Si concentration determined from the simulation of ellipsometric spectra by Tauc-Lorentz model. It can be concluded that the parameter 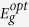 for AM films increases from 0.39 to 0.55 eV with the Si concentration rising to 33%. The broadening of 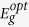 by Si doping can be explained as follows: the *a*-SST can be regarded as *a*-(Si + ST), based on the Vegard’s law, 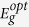 can be estimated by the following equation^36^:


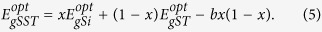


Here, *x* is Si concentration and *b* is the bowing parameter. Thus, the enlarged 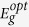 for SST films is due to the larger optical band gap of *a*-Si (∼1.7 eV) compared with *a*-ST. The relevant model of electronic band structure is given to explain and visualize the Si doping effects, as shown in Fig. 4(e). The ST in amorphous structure is a *p*-type semiconductor with an optical band gap of about 0.39 eV. The concentration of hole is larger than that of electron. The Fermi energy level (*E*_*F*_) is closer to the top of valence band. Besides, the *E*_*F*_ of *a*-Si is located at or near the middle of the forbidden band. For *a*-SST, the electronic band structure is similar with ST, it is still a *p*-type semiconductor with a larger optical band gap than ST. It is noteworthy that a sufficiently wide electronic band-gap is required for the sake of reducing the threshold current^37^. Thus, due to the larger 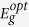, a lower threshold current can be obtained in the SST based phase-change memory cell.

**Figure 5:**
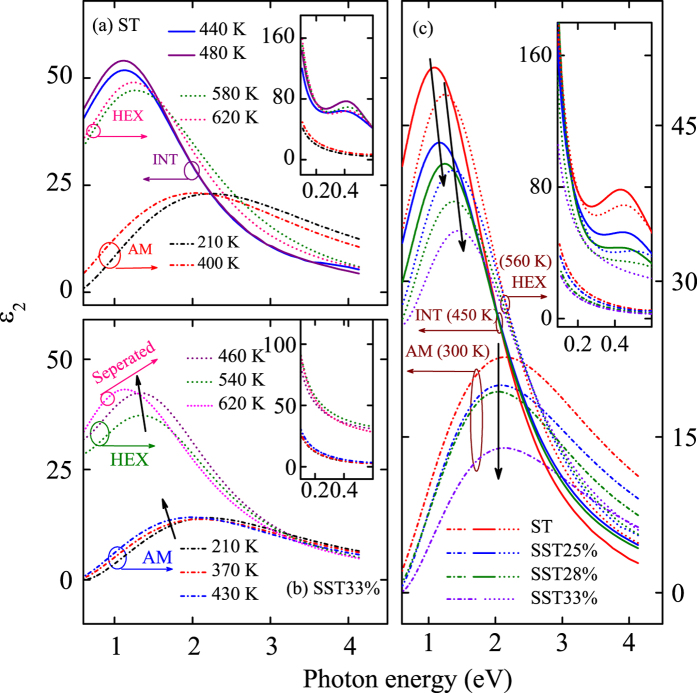
Evolutions of the imaginary part (ε_2_) for (**a**) ST and (**b**) SST33% films at several typical temperatures in the UV-vis region of 0.6–4.13 eV, respectively. (**c**) Evolutions of the imaginary part (ε_2_) for ST and SST films in AM (300 K), INT (450 K), and HEX (560 K) structures with different Si concentration in UV-vis region. Note that the insets show the corresponding ε_2_ in the IR region of 0.1–0.6 eV.

The imaginary part (ε_2_) of ST and SST33% at several typical temperatures are shown in Fig. 5(a,b), respectively. An obvious narrowing, redshift, and large enhancement of the main peak from amorphous (semiconductor) to crystalline (semimetal) structure can be observed. In addition, a striking Drude peak enhancement in IR region after crystallization can be found (insets of Fig. 5(a,b)). The tremendous contrast of dielectric functions between amorphous and crystalline structures is due to the order degree increment for crystalline phase^34^,^35^. Note that the gradual redshift of dielectric functions with increasing temperature is mainly associated with the electron-phonon interaction and lattice thermal expansion^38^. While for crystalline ST, the ε_2_ shows an obvious blueshift at about 520–540 K, which can be attributed to the change from INT to HEX state. The analogous phase change behavior also can be observed in SST25% and SST28% films. Furthermore, the monotonically redshift of ε_2_ is found in crystalline SST33% film. It indicates that the phase changes from AM to HEX structure directly, and then phase segregation appears with further heating up, based on the Raman spectra. Furthermore, the weak peak for ε_2_ in IR region of ST corresponds to the interband transition due to the band gap narrowing after crystallization or other intraband transition. The ε_2_ for ST and SST films in AM (300 K), INT (520 K), and HEX (560 K) structures with different Si concentration are shown in Fig. 5(c). The peaks show an obvious broaden, blueshift, and weaken with increasing Si concentration for crystalline films. The blueshift can be attributed to increasing interband transition energy by Si doping. The Si atoms still hold amorphous phase in crystalline films, which can be considered as the reason for the broaden and weaken, by leading to an increment of disorder degree^21^. However, the vertical position of the peaks is almost invariably for AM structure, which can be concluded that Si doping has little effect on the order degree for AM state. Note that the INT structure cannot be summarized for SST33% film, which is in accordance with the results from Raman scattering.

**Figure 6:**
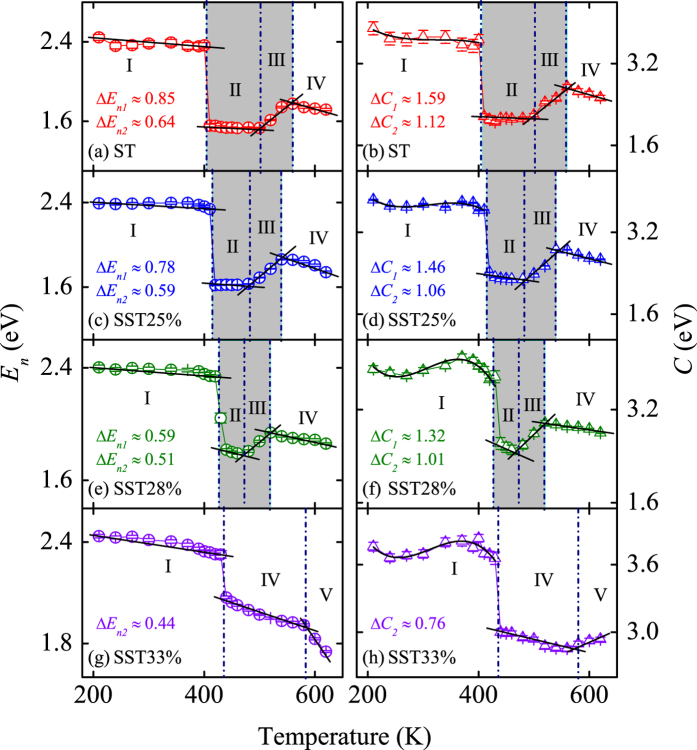
Evolutions of peak transition energy (*E*_*n*_) and the corresponding broadening terms (*C*) for ST and SST films with different Si concentration as a function of temperature. Note that the shadow regions correspond to the intermediates and transition states.

Next, the thermal evolutions of interband electronic transition energies (*E*_*n*_) and the corresponding broadening terms (*C*) for ST and SST are plotted in Fig. 6, which can be separated into five parts. Part I to V correspond to AM, INT, INT + HEX, HEX, and separated phase, respectively, based on the preamble relevant discuss. The phase change temperatures agree well with the result of Raman scattering. The sum of widths for Part II and III can be estimated to 150, 120, 90, and 0 K, corresponding to ST, SST25%, SST28%, and SST33% films, respectively. The narrowed intermediates and transition states can be related to the acceleratory crystallization of HEX phase by Si doping. The parameters Δ*E*_*n*1_ and Δ*C*_1_ are the change of *E*_*n*_ and *C* between AM and INT states, respectively. In similar, Δ*E*_*n*2_ and Δ*C*_2_ are that of the change between INT and HEX geometries. The values of Δ*E*_*n*1_, Δ*E*_*n*2_, Δ*C*_1_, and Δ*C*_2_ decrease from 0.85 to 0.59, 0.64 to 0.44, 1.59 to 1.32, and 1.12 to 0.76 with Si concentration rising to 33%, respectively. The decrease of Δ*E*_*n*_ corresponds to the peak blueshift in crystalline state by Si doping discussed in Fig. 5(c). Furthermore, the decrease of Δ*C* can be attributed to the reduced order degree discrepancy between amorphous and crystalline phases with increasing Si concentration.

**Figure 7:**
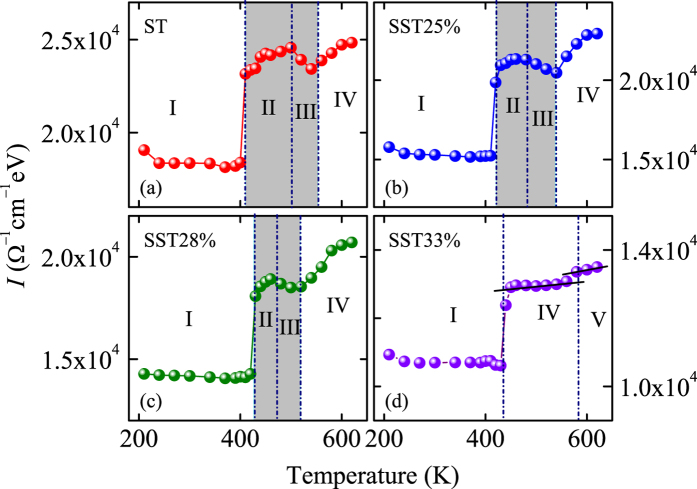
Partial spectral weight integral (*I*) evolutions of the ST and SST films in the photon energy region of 0.1–4.13 eV. (**a**) ST, (**b**) SST25%, (**c**) SST28%, and (**d**) SST33% as a function of temperature. Note that the shadow regions correspond to the intermediates and transition states.

To further verify the narrowed intermediates and transition states by Si doping between AM and HEX phases, the partial spectral weight integral (*I*) evolutions in the photon energy region of 0.1–4.13 eV are shown in Fig. 7. The parameter *I* reflects the electrons excited by photons during the selected energy range. It can be defined as^39^:


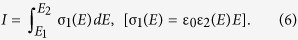


Here, σ_1_(*E*) is the optical conductivity, *E*_1_ and *E*_2_ are the lower and upper bound of the energy range, respectively. The evolutions of partial spectral weight integral as a function of temperature also can be separated into five parts (Part I to V), which is corresponding to the result of the change of interband electronic transition energies (Fig. 6). The parameter *I* near the first phase change temperature increases sharply, which corresponds to the phase change from AM to INT structure. It can be attributed to the strong optical absorption during the crystallization process. With further heating up, the partial spectral weight integral first increases slowly and then decreases to a minimum value, then it continues to increase. The temperature discontinuities of the variation trend can be associated with the phase change from INT to HEX phase for ST, SST25%, and SST28% films, as shown in Fig. 7(a–c), respectively. Besides, it can be concluded that the temperature range of intermediates and transition states is narrowed. For SST33% film, The parameter *I* increases monotonically with the elevated temperature, as shown in Fig. 7(d). Based on the discussions of the Raman spectra and dielectric functions, it can be summarized that the INT state disappears in SST33% films. The enhanced crystallization behaviors is demonstrated once again by the partial spectral weight integral.

## Conclusions

In summary, the lattice dynamics, optical constants, and electronic transitions of SST have been investigated by temperature dependent Raman scattering and SE experiments. The disappearance of intermediate state of SST33% film has been identified by the change of Raman phonon modes and dielectric functions. It illustrates that the possibility of phase separation (Sb and Te) decreases with the increasing Si concentration during the cyclic phase-change processes. The enhanced crystallization behaviors can improve the data retention ability. That is to say, the optimized data retention ability and the long term stability of ST by Si doping have been analyzed qualitatively from the optical perspective.

## Methods

### Fabrication of SST films

The SST films were deposited on SiO_2_/Si (100) via cosputtering pure stoichiometric Sb_2_Te and Si targets with a thickness of about 100 nm at room temperature. Meanwhile, in order to determine the Si doping concentration, the SST films were deposited on sapphire substrates at the same preparation conditions. The Si concentrations have been confirmed with the aid of Energy dispersive spectrometer (EDS), which are around 25, 28, and 33% (named SST25%, SST28%, and SST33%, respectively). Correspondingly, the pure ST film with the similar thickness was grown for comparison.

### Structural and optical characterizations

Raman spectra have been collected on heating by a micro-Raman spectrometer with a resolution better than 1 cm^−1^ (Jobin-Yvon LabRAM HR 800) and a THMSE 600 heating/cooling stage (Linkam Scientific Instruments) with liquid nitrogen as cooling accessories at a fixed heating rate of 10 K/min in the temperature range of 210–620 K. The Ar^+^ laser with the wavelength of 488 nm at a power of 5 mW was taken as the exciting source. The laser beam was focused through a 50× microscope with a working distance of 18 mm. An air-cooled charge coupled device (CCD) (−70 °C) with a 1024 × 256 pixels front illuminated chip was utilized to collect the scattered signal dispersed on 1800 grooves/mm grating. The surface morphologies of ST and SST33% films at several typical temperatures were recorded through the 50× microscope during the heating process in Raman scattering experiment. The temperature dependent SE measurements were performed in the photon energy range of 0.1–4.13 eV (300–12400 nm) at an incident angle of 70° by a vertical variable-angle SE (J. A. Woollam Co., Inc.). The spectral resolution is set to 10 nm, and the measurements were carried out with auto retarder (high accuracy). The samples were mounted into an Instec cell and Janis CRV-217V with liquid nitrogen as cooling accessories for high and low temperature experiments, respectively. The temperature is various from 210 to 620 K with a precision of about ±1 K. Note that the window corrections were included as a part of the model during the fitting analysis.

## Additional Information

**How to cite this article**: Guo, S. *et al*. Enhanced Crystallization Behaviors of Silicon-Doped Sb_2_Te Films: Optical Evidences. *Sci. Rep.* **6**, 33639; doi: 10.1038/srep33639 (2016).

## Supplementary Material

Supplementary Information
