# Supplementary material for: Enhanced Crystallization Behaviors of Silicon-Doped Sb2Te Films: Optical Evidences
Source: Sci Rep. 2016 Sep 19;6:33639. doi: 10.1038/srep33639 (PMC5027526; doi:10.1038/srep33639)
Supplement: Supplementary Information [file srep33639-s1.pdf]

Supplementary Information

Enhanced Crystallization Behaviors of Silicon-Doped Sb_2Te Films: Optical Evidences

Shuang Guo[1], Liping Xu[1], Jinzhong Zhang[1], Zhigao Hu[1,*], Tao Li[2], Liangcai Wu[2], Zhitang Song[2] & Junhao Chu[1]

[1]Department of Electronic Engineering, East China Normal University, Shanghai 200241, China.
[2]State Key Laboratory of Functional Materials for Informatics, Shanghai Institute of Microsystem and Information Technology, Chinese Academy of Sciences, Shanghai 200050, China.

[*] Corresponding author. Tel.: +86-21-54345150. Fax: +86-21-54345119.
Electronic mail: zghu@ee.ecnu.edu.cn

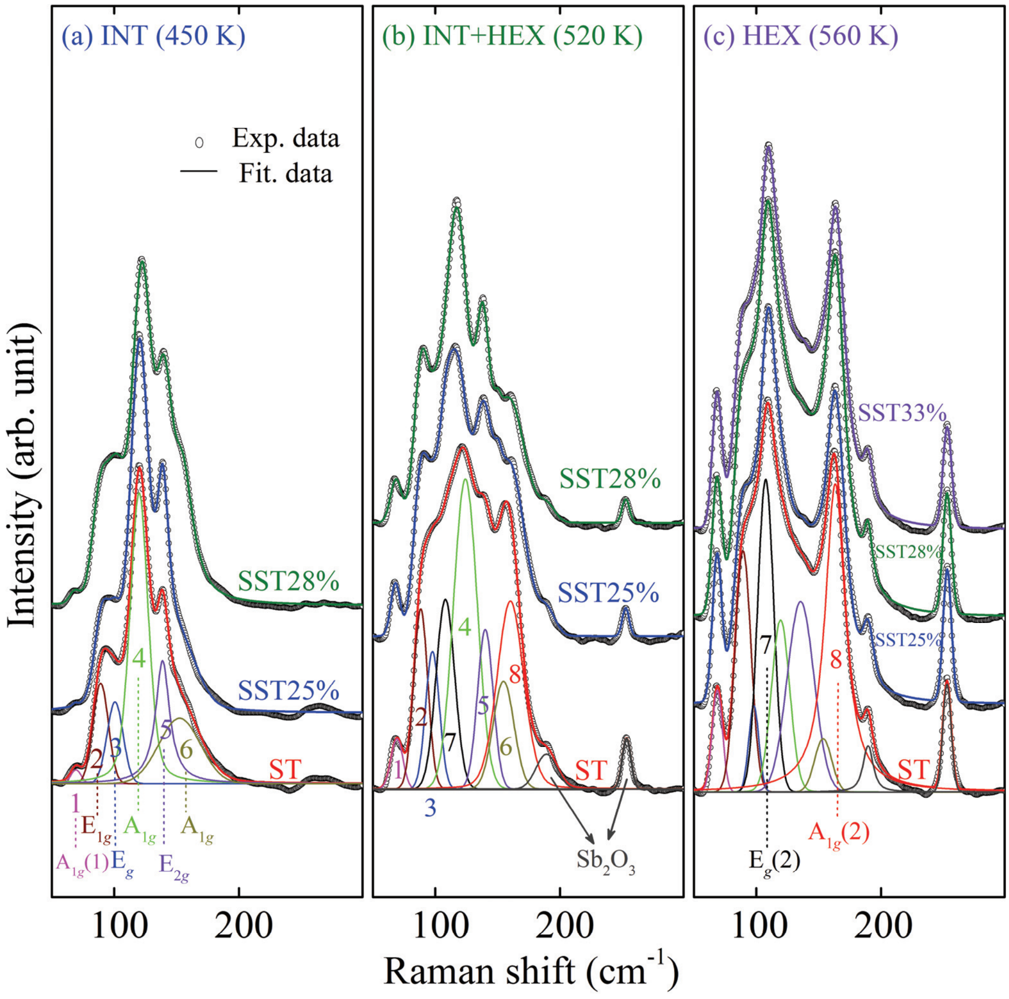

Fig. S1 Raman spectra of ST and SST at (a) 450, (b) 520, and (c) 600 K with different Si concentration, respectively . Note that the fitting to the Lorentz-Gaussian functions for ST is given as an example, and the explanatory note of these Raman phonon modes are presented.

Table S1: The assignments of phonon modes from fitting Lorentz-Gauss oscillators and the frequencies of each oscillator are recorded at 450 K (INT) and 560 K (HEX). Note that the unit of the frequencies is cm^{-1} and the error bars are presented in parentheses.

Geometries	peak-number	Raman modes	ST	SST25%	SST28%	SST33%
INT	1	$A_{1g}(1)$ (Sb-Te)	68.4 (1.44)	68.6 (0.92)	68.8 (0.56)	-
	2	E_{1g} (Te-Te)	89.2 (2.42)	89.1 (1.07)	89.8 (0.79)	-
	3	E_g (Sb-Sb)$_g$	100 (3.16)	101 (1.13)	102 (0.59)	-
	4	A_{1g} (Te-Te)	120 (0.20)	120 (0.08)	122 (0.16)	-
	5	E_{2g} (Te-Te)	138 (0.23)	139 (0.10)	139 (0.20)	-
	6	A_{1g} (Sb-Sb)	152 (0.83)	154 (0.66)	155 (0.71)	-
HEX	1	$A_{1g}(1)$ (Sb-Te)	68.5(0.11)	68.6 (0.07)	68.6 (0.09)	68.7 (0.08)
	2	E_{1g} (Te-Te)	89.1 (0.57)	89.4 (0.64)	89.6 (0.65)	89.8 (0.52)
	3	E_g (Sb-Sb)	98.0 (0.21)	98.5 (0.39)	98.5 (0.20)	98.8 (0.19)
	4	A_{1g} (Te-Te)	118 (0.51)	119 (0.64)	119 (0.43)	120 (0.34)
	5	E_{2g} (Te-Te)	136 (0.97)	137 (0.96)	137 (0.16)	138 (0.44)
	6	A_{1g} (Sb-Sb)	149 (1.22)	150 (0.81)	151 (0.96)	152 (0.23)
	7	$E_g(2)$ (Sb-Te)	108 (0.94)	109 (0.79)	109 (0.78)	110 (0.21)
	8	$A_{1g}(2)$ (Sb-Te)	162 (0.47)	163 (0.65)	164 (0.41)	164 (0.58)

Table S2. Dielectric function parameters of the Tauc-Lorentz, Lorentz, and Drude oscillator models for ST and SST films are determined from the simulation of ellipsometric spectra at 300 (AM), 450 (INT), and 560 K (HEX), respectively. The "σ" indicates root-mean-square fractional error for the fitting. Note that the 95% reliability of the fitting parameters is given in parentheses. All the parameters are in eV units.

Sample		ST		SST25%		SST28%		SST33%	
Regions		IR	UV-vis	IR	UV-vis	IR	UV-vis	IR	UV-vis
300 K	A	6.53 (0.53)	126 (0.13)	4.51 (0.62)	120 (0.33)	3.65 (0.23)	98.5 (0.21)	3.28 (0.96)	86.7 (0.85)
TL(L)+Drude	C	0.39 (0.22)	3.72 (0.13)	0.15 (0.54)	3.76 (0.01)	0.09 (0.33)	3.87 (0.05)	0.55 (0.33)	3.69 (0.04)
	E_n	0.71 (0.12)	2.38 (0.03)	0.69 (0.14)	2.39 (0.01)	0.70 (0.17)	2.39 (0.01)	0.16 (0.02)	2.41 (0.01)
	E_g^{opt}	-	0.39 (0.01)	-	0.46 (0.01)	-	0.50 (0.01)	-	0.55 (0.01)
	A_d	4.69 (0.77)	-	4.27 (0.48)	-	2.88 (0.61)	-	1.74 (0.54)	-
	Br_d	0.60 (0.34)	-	0.61 (0.50)	-	0.70 (0.08)	-	0.17 (0.07)	-
	σ	5.06	0.83	5.17	1.64	3.59	0.04	5.95	2.17
450 K	A	65.6 (0.15)	41.6 (0.22)	40.1 (0.81)	34.1 (0.45)	37.7 (0.88)	36.5 (0.13)	-	-
Lorentz+Drude	C	0.46 (0.02)	2.13 (0.02)	0.50 (0.04)	2.29 (0.01)	0.41 (0.01)	2.55 (0.01)	-	-
	E_n	0.50 (0.02)	1.53 (0.01)	0.55 (0.03)	1.61 (0.01)	0.47 (0.01)	1.80 (0.01)	-	-
	A_d	16.6 (0.19)	8.25 (0.20)	15.8 (0.91)	7.21 (0.29)	15.1 (0.48)	5.36 (0.17)	-	-
	Br_d	0.25 (0.01)	0.82 (0.15)	0.31 (0.01)	0.84 (0.26)	0.30 (0.01)	0.71 (0.07)	-	-
	σ	3.14	0.24	4.87	0.51	3.96	0.15	-	-
560 K	A	57.2 (0.78)	42.6 (0.27)	46.2 (0.21)	36.8 (0.22)	40.1 (0.10)	35.1 (0.11)	37.5 (0.75)	20.4 (0.07)
Lorentz+Drude	C	0.52 (0.02)	2.75 (0.04)	0.64 (0.06)	2.86 (0.01)	0.47 (0.02)	2.91 (0.01)	1.22 (0.03)	2.85 (0.02)
	E_n	0.54 (0.02)	1.78 (0.01)	0.64 (0.03)	1.86 (0.01)	0.70 (0.01)	1.90 (0.01)	0.17 (0.12)	1.92 (0.01)
	A_d	19.3 (0.28)	11.6 (0.44)	18.2 (0.62)	9.58 (0.26)	17.9 (0.70)	8.31 (0.64)	12.3 (0.49)	5.02 (0.38)
	Br_d	0.23 (0.01)	0.45 (0.05)	0.29 (0.01)	0.54 (0.04)	0.28 (0.01)	0.47 (0.02)	0.12 (0.06)	0.21 (0.02)
	σ	5.39	0.42	4.22	0.40	3.59	0.21	3.42	0.13
